# Supplementary material for: Assessing insecticide susceptibility, diagnostic dose and time for the sand fly Phlebotomus argentipes, the vector of visceral leishmaniasis in India, using the CDC bottle bioassay
Source: PLoS Negl Trop Dis. 2023 May 10;17(5):e0011276. doi: 10.1371/journal.pntd.0011276 (PMC10202287; doi:10.1371/journal.pntd.0011276)
Supplement: Attachment — Submitted filename: P argentipes insecticide Reviewer Response.doc [file pntd.0011276.s001.doc]

February 8, 2023

Jean-Philippe David, PLOST NTD Academic Editor, and Alvaro Acosta-Serrano, PLOS NTD Section Editor:

I would like to express my appreciation for the opportunity to address some of the concerns identified by the peer reviewers specific to the manuscript titled “**Assessing insecticide susceptibility, diagnostic dose and time for the sand fly *Phlebotomus argentipes,* the vector of visceral leishmaniasis in India, using the CDC bottle bioassay**”. We believe the research findings provide a valuable technical resource for the readership of PLOS NTD. The reviewers identified several concerns specific to the writing in the manuscript. I believe that we have addressed these concerns in the edited manuscript.

A detailed account of how each modification and concern has been addressed and revised in the manuscript. This account can be found at the bottom of this letter. The reviewer’s concern was initially written out and the author’s response to each concern can be found, in red, directly under the concern. We hope that we have effectively addressed these concerns and look forward to having this manuscript published in PLOS NTD. Please don’t hesitate to contact me with any questions.

Sincerely,

Scott A. Bernhardt, Ph.D., M.P.H.

Utah State University

Department of Biology

UMC5305 Old Main Hill

Logan, Utah 84322

(435) 797-3721

[scott.bernhardt@usu.edu](mailto:scott.bernhardt@usu.edu)

How the author’s addressed each of the modifications and reviewer concerns:

**Major modifications:**

- please correct all typo errors (word spacing, section titles, bibliography formatting, ...).

The authors reviewed the manuscript and believe the current version being submitted has been “cleaned” up.

- As the data presented may be used as a baseline by subsequent studies, the insecticide susceptibility profile of the laboratory strain used in the present study should be made cleared in the method section (reference #36 does not report resistance data/status) and supported by ANY available data. Defining insecticide diagnostic doses usually implies testing multiple 'susceptible' strains of distinct geographical origins to be sure that the diagnostic dose proposed is coherent. This point is crucial important as the presence of DDT (and PYR?) resistance allele is highly suspected in the line used for the present study... This should also be further discussed in the discussion section (e.g. comparison with other studies).

Wild sand flies were collected in 2015 from the village in which residual spraying of DDT had not been done within 1 or 2 years (No VL cases reported from 2013 onward) before collection and IRS using synthetic pyrethroids was started in 2016. Closed colony flies were not exposed to any insecticide and after 20^th^ generation started this study.

The authors appreciate the reviewers highlighting the need to test multiple ‘susceptible

Strains of distinct geographical origins. In order to evaluate multiple geographical susceptible strains, it is critical that baseline susceptibility and diagnostic doses are determined before this evaluation. This is what this manuscript is demonstrating – the baseline data that can then be used to evaluate field strains. The methods and findings are set up in similar fashion to Denlinger et. al. 2016. As described in the discussion, the next phase of the research is we are actually evaluating resistance in field populations in Northern India and look forward to publishing these findings after this manuscript.

- In the results section, I am not sure it is necessary to make a whole paragraph comparing LC50s from different insecticides as different molecules have different inherent toxicity.

The authors agree with this reviewer’s suggestion and have eliminated aspects of this paragraph.

**Minor modifications:**

- Line 215: the definition of "diagnostic dose" proposed is not the one used by WHO (which is usually twice the dose killing 100% of susceptible individuals).

The authors appreciate the reviewer’s recognition of the definition of “diagnostic dose” and the what is used by the WHO. As the reviewer stated and/or is referring to “the concentration corresponding to 99.9% mortality; at this concentration there is a high probability that all individuals in a susceptible population will be killed. Double this concentration is conventionally known as the discriminating (or diagnostic) concentration.”

The authors need to note that the research findings in this manuscript is based on using CDC bottle bioassay (Brogdon W, Chan A. Guideline for evaluating insecticide resistance in vectors using the CDC bottle bioassay. USA: CDC Atlanta. 2010). The WHO bioassay measures mortality rates in vectors exposed to a discriminating concentration of insecticide for a fixed period of time. Whereas, the CDC bottle bioassay measures a discriminating (diagnostic) length of time required to incapacitate susceptible vectors using a predetermined concentration of insecticide.

- line 320: I do not agree about the statement of different biochemical target between PYR and DDT, modify or provide a solid reference to support this statement.

The authors appreciate the reviewers concern about how the biochemical targets have been described. We have gone in and added a reference in the text. Both DDT and pyrethroids target the insect VGSC protein. DDT increases sensitivity for depolarization of the channel, while pyrethroids only inhibit inactivation/deactivation processes of the channel; nevertheless each leads to stabilization of the open state and repetitive nerve firing, paralysis and ultimately death (Gomes et al. 2017). DDT affects mainly the peripheral nervous system, initial contact with the insecticide causing neurons to fire spontaneously causing muscles to twitch, with resulting tremors throughout the body and appendages, the so-called ‘DDT jitters’. Eventually, over the course of a few hours or days, DDT exposure leads to excitatory paralysis and consequent death of the insect. Compared with some insecticides DDT is rather slow acting. (Davies et al. 2007). The pyrethrin and pyrethroid insecticides affect both the peripheral and central nervous systems of insects. They initially stimulate nerve cells to produce repetitive discharges and eventually cause paralysis, an effect similar to, but more pronounced than that of DDT (Davies et al. 2007).

- line 378-380: tone down. before being used as a reference, the doses and times reported in the present study should be compared with data obtained from other lines of the same species.

The authors have gone and modified this paragraph. In absence of sand fly specific WHO susceptibility test procedure and impregnated papers, all phenotypic bioassays with wild sand flies are conducted with WHO susceptibility test kits only except few which are discussed. We are using the CDC bottle bioassay to measure the susceptibility and diagnostics doses, and recognize that the results of the two bioassays methods may not be directly comparable as CDC bottle assays measure knockdown at the end of a predefined time, whereas WHO test kit bioassays measure mortality at 24 hours or 72 hours for slow acting compounds.

**REVIEWER #1**

Manuscript submitted by Chaubey et al. is describing the resistance profile and experiments about insecticide. The subject of this manuscript is within the field of interest of the Journal and the methods are appropriate and well described. The paper is well \written and concise and, as such, is suitable for publication in PNTD. The literature is well reviewed, experimental design and statistical methods are adequate. There are some specific points recommendations.

Line 50: correct "insectcidesare" – Correction made

Line 51: correct " Sand flieshad" – Correction made

Line 56: correct "timesare" – Correction made

Line 68: correct "Leishmaniadonavani" – Correction made

line 73: correct "Thisresting" – Correction made

Line 79: correct "leishmaniasisresurged" – Correction made

There are also other typos that authors should check trough the manuscript. – Manuscript reviewed by several individuals to identify and correct typos.

Line 180: I dont understand why specimens were released to bottles with mixed genders? Why both sexes did not tested separately?

In nature both male and female live in the same vicinity and have similar potential for being exposed to insecticides. Various population dynamic studies published studies capture both the sexes from houses, cattle sheds and other dwellings and demonstrate that they are both exposed to insecticides. To maintain the natural “neighborhood”, we used both sexes and released them in the same bottle. We are not certain why the reviewer is suggesting why sexes should be separated and evaluated separately for insecticide susceptibility.

Line 185: I think there should be a standard exposure time? Why its 30 mins or 120 mins.? Also, did 30 mins results and 120 mins results were analyzed together?

Excellent question! As described in Brogdon W, Chan A. Guideline for evaluating insecticide resistance in vectors using the CDC bottle bioassay. USA: CDC Atlanta. 2010, 30 minutes is a standard exposure time for all insecticides, except for DDT. The reason why the 30 minutes standard time was adjusted for DDT had to do with sand fly survivability to exposed concentration. At the 30-minute time exposure to DDT, nearly 100% of all sand flies survived. Preliminary data collections consisted of increasing DDT concentration, but the concentration was so high that it was unrealistic as to what would even be used in the field. After reviewing Brogdon W et. all 2010, the more appropriate approach was to adjust the exposure time to 120 minutes, depending on expected and actual survival rates. All the results were analyzed separately.

**Reviewer #2:**

Mechanical aspirator used for collection of sandflies that causes the damage of sandflies that may enhanced the mortality of the exposed sandflies.

The concern described by reviewer 2 about mechanical aspiration is a valid. Substantial training occurs with student researchers to teach how to avoid and limit damage to sand flies while moving from one container to another. If by chance there is mechanical damage during the transfer process in a replicate, the damage can be reasonably accounted for since aspiration is performed by the same individual for all the bottles in the same replicate. Flies are placed in one control bottle in every replicate and mortality correction was done by using Abbott’s formula.

Only one timer used for four bottles, but for accurate time maintenance four timers should use.

Excellent catch in the text – In all reality, a separate timer was actually kept for each bottle to maintain time accuracy. The text has been modified to relate how timing was actually performed.

Line 261-262: Reference should not mention in the result, however it may move to 'discussion' section.

Reference cited was removed and sentence modified

Line 312: Insecticides tested against sandflies, but mentioned different disease vectors, the statement is confusing.

Statement has been deleted from the text

Line 310-315: Should be deleted.

Agreed - complete paragraph was deleted

Line 339: What is Deb et al. 2021?

This has been deleted from the text

How findings of the study will facilitate the "Indian kala-azar elimination program" need to mention at the end of the manuscript (conclusion).

More descriptive conclusions have been added to the text

Reviewer #2: All issues are addressed except ethical concern, even not mentioned anything about the regulatory authority who had approved the proposal for conducting the study.

An ethics statement has been added

Reviewer #2: Not drawn any appropriate conclusion.

Not discussed the strength and weakness of the study.

Not mentioned the public health relevance of the study.

A descriptive conclusion, potential limitations, and public health relevance writing has now been added to the discussion.

**Reviewer #3:**

It's a useful study that describes the insecticide susceptibility pattern of sand flies in a laboratory-bred colony in India, which will provide useful information by way of baseline data, which is lacking.

The tables and figures should contact legends to enable easy interpretation by the readers and abbreviated terms should be spelt out.

The authors are a little puzzled about the request from reviewer #3 about the tables and figures needing legends to improve interpretation. Table 1 has subheadings in the table that lists out the insecticide, concentration, and time. Table 2 has subheadings in the table that lists out the insecticide and the lethal concentration that kills 50, 90, and 95% with a lower and upper confidence limit. Table 3 has subheadings in the table that lists out the insecticides, diagnostic dose concentration, and diagnostic time-to knockdown and 24-hour mortality. Figure 1 shows the susceptibility curves with all the replicates (dots) for each insecticide with percent mortality on one axis and insecticide concentration on the other axis. The authors would be happy to provide additional information in the figures/tables, but are not certain what else could be added.

More recent publications on insecticide susceptibility in the region should be added to the discussion.

Additional publication references have been added, but it is important to note that there is limited work that has been conducted in this region of India.

Reviewer #3: Yes in large part. The numbers of sand flies tested should be given in the abstract.

The authors are uncertain what the reviewer is requesting specific to number of sand flies tested in the abstract. The approximate number of sand flies (20-30) tested in each bottle replicate was/is written out in the abstract, as well as in the methods. Is the reviewer requesting that we describe the total number of flies test overall – or for each concentration?

Reviewer #3: Though the methods section says the experiments were repeated 3 times, that information isn't shown in the results section.

None of the figures or tables have legends, therefore difficult to interpret.

The authors appreciate the concern brought up by reviewer #3, but are a bit puzzled at how to respond and/or edit the manuscript. As highlighted by the reviewer, the methods section says the experiments were repeat at minimum 3 times. But then there was a concern about this information not being described in the results. The authors didn’t think it was necessary to re-describe the methods process in the results section. One though, can see in Figure 1 that multiple replicates (a minimum of 3) were conducted for each concentration. When it does look like one “dot” or concentration replicate, the mortality rate for each replicate at the concentration was nearly uniform.

As for the figures and tables not having legends, an explanation is described above. The authors are puzzled at what additional informative language could be included in a legend.

The abbreviated terms used in the text and in illustrations should be described properly e.g. 24 hour mortality

The authors have revised the text to include written out descriptions prior to using an abbreviated term.

Reviewer #3: Yes, in large part.

However, the conclusions could be toned down and viewed against the existing information from the region. It'll be useful to access more recent publications on insecticide susceptibility studies in the South Asian region to discuss the results more meaningfully.

Limitations of the studies also could be added.

The authors appreciate the recommendation made by the reviewer concerning the conclusions. Sections of the discussion/conclusion have been edited. Additional language has been added and citations have been revised.
