# Supplementary material for: Assessing insecticide susceptibility, diagnostic dose and time for the sand fly Phlebotomus argentipes, the vector of visceral leishmaniasis in India, using the CDC bottle bioassay
Source: PLoS Negl Trop Dis. 2023 May 10;17(5):e0011276. doi: 10.1371/journal.pntd.0011276 (PMC10202287; doi:10.1371/journal.pntd.0011276)
Supplement: Attachment — Submitted filename: Comments-1.docx [file pntd.0011276.s002.docx]

**Comments**

The authors are supposed to submit a revised version of the manuscript highlighting the track change mode which is missing; therefore, it is difficult to find out where changes have been made. It is one of the requirements of the journal.

Line 83: After *P. argentipes*, mention “sandfly” throughout the manuscript.

Line 86-87: Indian national malaria program, mention it as “Indian national malaria eradication program”

Line 90: in 2003, it will be “2005” as the Regional VL elimination programme was launched in 2005.

Figure-2: Need to revisit the study findings to confirm it is correctly presented.

Line 364: need to correct - (WHO, 1986,[27, 41-44].

Line 375-376: …….. lower concentration (Figure 1 and Table 2). Any figure or table of the results should not directly refer to the ‘discussion’ section.
